# Genetic Differences in Purple Finch (*Haemorhous purpureus*) Are Linked With Subspecies and Migratory Behaviour Differences

**DOI:** 10.1002/ece3.73650

**Published:** 2026-05-08

**Authors:** Danika Schramm, Theresa M. Burg

**Affiliations:** ^1^ Department of Biological Sciences University of Lethbridge Lethbridge Alberta Canada

**Keywords:** adaptation, migration, population genomics

## Abstract

Seasonal migration patterns and geological barriers can impact population structure. In particular, migratory behaviour (resident vs. migratory) differences are thought to promote genetic structure within species. Using a ddRADseq approach, we genotyped 43 purple finches ( *Haemorhous purpureus* ) from eight populations across their range comprising both subspecies and examined the effect of migratory behaviour on population structure in this species. We also used genome scans to identify loci under selection and tested whether these differences are associated with migratory behaviour differences. The two subspecies were genetically distinct and within *H. p. californicus*, we found that populations formed three genetic clusters. Population structure was not strongly associated with resident or migratory behaviour, although we did identify five regions under selection that were distinct between migratory and resident populations. Two of the genes identified in these scans were related to ghrelin hormone and liver fat deposit; Ghrelin is a key component of migratory restlessness, while liver fat deposits are used to fuel migration in birds. Combined our results suggest that population genetic differences between subspecies reflect historical isolation during the Last Glacial Maximum, and indicate that some genetic differences occur between resident and migratory populations. These genetic differences appear to be associated essential for pre‐migratory and migratory behaviour in birds.

## Graphical abstract

We used ddRADseq data to assess the genetic population structure of purple finch populations in North America. We found differences among and within subspecies. We also found genes that were under selection and associated with differences observed in subspecies and migratory phenotypes.

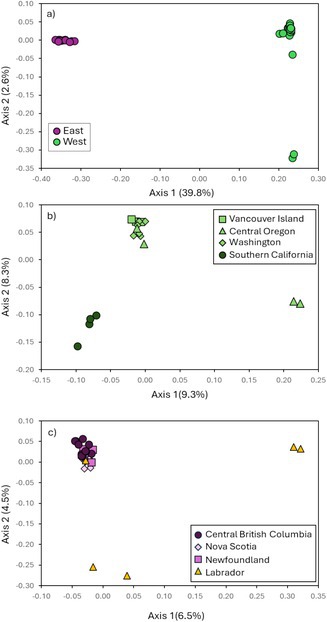

## 1 Introduction

Seasonal migration is a highly studied behaviour and can have important evolutionary implications. The underlying genomic cause for this complex behaviour is species dependent and complicated by geological features such as mountains. Migratory behaviour facilitates greater levels of gene flow in comparison to resident populations (Delmore et al. 2020; Ruegg et al. 2014), and use of different migration routes can result in population divergence (Gu et al. 2021; Lundberg et al. 2017). Multiple genes appear to be responsible for migration, and the genes involved vary between species (Delmore et al. 2020, 2016; Lundberg et al. 2017; Parody‐Merino et al. 2019; Peterson et al. 2013). As a result, it is critical to use ecological and genomic approaches to study behavioural differences between populations.

Genetic isolation can result from behavioural differences or from breaks in suitable habitat in historical or contemporary time periods. Contemporary breaks in habitat can appear in the form of mountains, lakes, rivers and habitat fragmentation. Differences in genetic structure can arise even when breaks in habitat are relatively small (Adams and Burg 2015; MacDonald et al. 2020; Perrin et al. 2020). In the Northern Hemisphere, many species show population structure due to the impacts of the most recent glaciation (Pielou 1992; Weir and Schluter 2004). High latitude species were forced to remain on the peripheries of the ice in glacial refugia (Hewitt 1996, 2000, 2004). If a species resided in multiple glacial refugia, this extended period of isolation often resulted in genetic structure that is still observed after the ice sheets have retreated (Hewitt 1999; Burg et al. 2006, 2014; Knowles 2001; Macfarlane et al. 2016; Weir and Schluter 2004).

**FIGURE 1:**
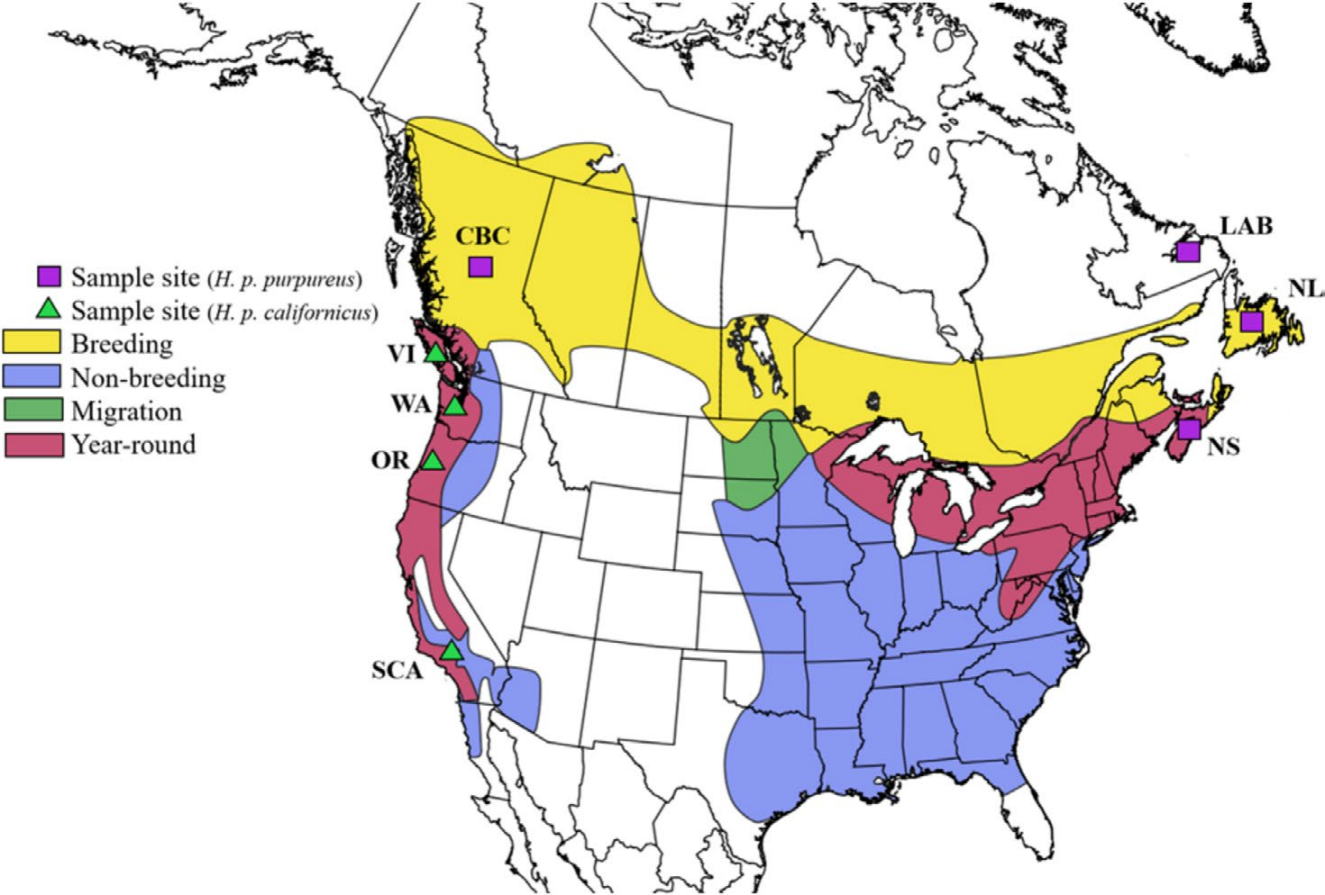
Range map and sampling locations of the two purple finch subspecies: *H. p. californicus* (green triangles) and *H. p. purpureus* (purple squares). Note that although Labrador appears outside of the typical range, purple finches are reliably captured and observed in the area. Additionally, Newfoundland populations are commonly listed as migratory and were considered such for this study. Range data obtained from Birdlife International and Handbook of the Birds of the World (2022), and map created in QGIS 3.2 (QGIS Development Team 2022).

The purple finch, *Haemorhous purpureus* , is a high latitude species that displays population structure related to the most recent glacial period (Macfarlane et al. 2016). This species primarily breeds in coniferous forests; however, it can breed in a range of forest types (Wootton 2020). In addition to its high latitude habitat, the purple finch has both resident and migrant populations, lending itself to being a study system for both behavioural and physical barriers to gene flow (Figure 1).

Previous studies on the purple finch analyzed their population genetic structure using mitochondrial DNA (mtDNA), microsatellite markers and Z‐linked sequences (Macfarlane et al. 2016). The two main genetic groups correspond to the two recognised subspecies: *H. p. purpureus* and *H. p. californicus* (Macfarlane et al. 2016). *H. p. purpureus* includes individuals breeding in central British Columbia and stretching east across to Newfoundland and south to West Virginia and migrate south to the eastern United States during winter months. *H. p. californicus* breeds along the Pacific Coast from British Columbia down to southern California and is primarily resident (Figure 1). In addition to the genetic differences, the two subspecies show morphological, plumage, behavioural and song differences that enable them to be distinguished reliably without the use of genetic data (Macfarlane et al. 2016; Wootton 2020).

Within the subspecies groups, Macfarlane et al. (2016) found additional genetic groupings that may be the result of various barriers to gene flow. Their microsatellite analyses detected up to six groups (OR/CA, WA, VI/CBC/LAB, MI/ND, NS and NL) and suggested secondary contact between the two subspecies may be occurring in British Columbia (Macfarlane et al. 2016). While it does not appear that physical barriers explain all observed structure, behavioural differences including migratory behaviour and site fidelity may influence the contemporary patterns observed (Macfarlane et al. 2016).

While the previous study provided a thorough basis for examining the genetic patterns of the purple finch, utilising restriction enzyme‐associated DNA sequencing (ddRADseq) will allow for genome‐wide sampling and the ability to identify loci that are putatively under selection. Genome scans comparing the subspecies as well as resident and migratory populations can shed light on some of the genes that influence phenotypic differences and genetic differentiation. Studies of other organisms have found genomic differences in resident and migratory populations as well as differences between migratory phenotypes (Delmore et al. 2020; Liedvogel et al. 2011; Lundberg et al. 2017; O'Malley et al. 2010; Pearse et al. 2014; Peterson et al. 2013; Toews et al. 2019). In this study, we examined whether migratory behaviour influences genetic structure by testing whether migratory and resident populations form distinct genetic clusters and conducted genome scans to detect potential loci under selection that might drive these migratory differences. Our present study expands on the previous study by Macfarlane et al. (2016) by using ddRADseq data to examine these questions. We predicted that resident populations are genetically distinct from migratory populations because of limited dispersal by resident birds. Further if migratory behaviour is driven by genetic differences, then we will detect putative loci under selection from genes associated with physiological or behaviour differences required for migration. Finally, we used genomic scans to detect genetic differences between subspecies to determine whether loci under selection drive the genetic differences between *H. p. purpureus* and *H. p. californicus* previously reported by Macfarlane et al. (2016).

## 2 Methods

### 2.1 Sample Collection

From 2007 to 2013, 43 purple finch from eight locations were sampled during the breeding season. These eight sample sites were southern California (SCA), coastal Oregon (OR), Washington (WA), Vancouver Island (VI), central British Columbia (CBC), Nova Scotia (NS), Newfoundland (NL) and Labrador (LAB) (Figure 1; Table 1). Birds were captured using song playback and mist nets. Once captured, birds were fitted with an aluminum band to ensure no bird was sampled more than once. A small blood sample (< 50 μL) was collected from the brachial vein and stored in ethanol. Captured birds were released at the capture site.

**TABLE 1:** Sample locations included in this study along with their subspecies and migratory classification designation, sample size (*n*) and observed (*H*_o_) and expected (*H*_e_) heterozygosity values from each population.

Sample site	*n*	Subspecies	Migratory phenotype	*H*_o_	*H*_e_
Southern California (SCA)	4	*H. p. californicus*	Resident	0.36	0.38
Coastal Oregon (OR)	4	*H. p. californicus*	Resident	0.38	0.38
Washington (WA)	6	*H. p. californicus*	Resident	0.29	0.31
Vancouver Island (VI)	3	*H. p. californicus*	Resident	0.41	0.43
Central British Columbia (CBC)	11	*H. p. purpureus*	Migratory	0.19	0.21
Nova Scotia (NS)	5	*H. p. purpureus*	Resident	0.29	0.32
Labrador (LAB)	5	*H. p. purpureus*	Migratory	0.40	0.42
Newfoundland (NL)	5	*H. p. purpureus*	Resident	0.29	0.32

*Note:* Sample site abbreviation codes are listed in brackets after the name of each population.

### 2.2 DNA Extraction and Sequencing

DNA extractions were performed using a modified phenol chloroform extraction protocol prior to being sent for restriction enzyme‐associated DNA sequencing (Sambrook and Russell 2006). Library preparation occurred at Plateforme d'Analyses Génomiques using the *PstI, MspI* and *NsiI* restriction enzymes (Abed et al. 2019). Prepared samples were sequenced on an Illumina NovaSeq 6000 25 M with paired‐end reads at Génome Québec.

### 2.3 Data Processing

Raw sequence reads were analyzed using Fastqc 0.11.5 to generate quality reports to ensure no major errors occurred during sequencing (Andrews 2010). Raw reads were demultiplexed using Sabre 1.00. Adapter and barcode sequences were removed using Cutadapt (Martin 2011). We also used Cutadapt to trim reads to 80 base‐pairs in length as Stacks 2.3e. Since a purple finch genome was not available, we used the house finch ( *Haemorhous mexicanus* ) genome (GCA_027477595.1, chromosome level assembly) because they are closely related to the purple finch. The reference genome was indexed using samtools 0.1.2 (Danecek et al. 2021) and the Burrows‐Wheeler Alignment Tool (bwa) aligned the forward and reverse reads (Li and Durbin 2010). SNP calling was performed using the Stacks 2.3e *ref_map* pipeline with the default parameters and ‐write‐single‐snp enabled. The resulting vcf file was filtered using VCFtools 0.1.16 to remove SNPs and individuals with more than 20% missing data. We checked all loci for linkage disequilibrium in Plink 1.9 (Chang et al. 2015) to avoid including any loci that violated this assumption. The aligned and filtered dataset contained 43 individuals and 76,154 SNPs.

### 2.4 Population Analyses

Mean observed and expected heterozygosities for each population were calculated in Arlequin 3.5.2.2 (Excoffier and Lischer 2010) along with pairwise *F*_ST_ and the corresponding *p*‐values. To account for multiple comparisons, the *p*‐values were corrected using the Benjamini‐Hochberg method (Benjamini and Hochberg 1995).

Pairwise Sforza Chord genetic distances were calculated between individuals using GenoDive and then used for Principal Coordinate Analysis (PCoA) in GenAlEx 6.5 (Peakall and Smouse 2006). We also compared genetic patterns within each subspecies with a PCoA to examine any within‐group differentiation.

An ancestry matrix was constructed using LEA, with the cross‐entropy function enabled (Frichot and Francois 2015). The program ran for 100 iterations with the number of assumed populations between one and six. LEA was also run on each subspecies individually to examine any within‐group differentiation. We complemented the LEA analysis by using Discriminant Analysis of Principal Components (DAPC) from the ADEGENET package (Jombart 2008) in R. DAPC converts genetic data to principal components and then uses *k*‐means clustering algorithms to identify the number of genetic clusters in the data. DAPC is often used as secondary analyses to other population genetic analyses like PCoA because it reduces within‐group variation to examine between‐group variation in greater detail. As such, it is more sensitive than many other approaches (Jombart 2008).

### 2.5 Genome Scans

We performed two sets of genome scans by calculating pairwise *F*_ST_ values across the genome between resident and migratory populations regardless of subspecies and then between the two subspecies groups (Danecek et al. 2011). Pairwise *F*_ST_ values were all calculated with VCFTools 0.1.16 using 100 kb sliding windows with a 100 kb step. Additionally, we calculated π (nucleotide diversity) in 100 kb windows with 100 kb steps within each of the four groups of interest. Manhattan plots for both *F*_ST_ and π were visualised in R‐studio using ggplot2 (Wickham 2016). We evaluated the 99.9th percentile of the windowed *F*_ST_ values in R studio and cross referenced *F*_ST_ values with the lowest and highest π values to determine areas that may be under selection or have experienced a recent selective sweep as these regions would appear similar (Ravinet et al. 2018). Chromosomes containing *F*_ST_ windows in the 99.9th percentile and elevated or depressed π values were pulled from the dataset for additional analyses as *F*_ST_ can be prone to biases and false positives, making it useful to perform additional analyses to confirm the presence of selection (Vitti et al. 2013).

Haplotype analyses were performed following a similar protocol to Ravinet et al. (2018). To prepare chromosomes for haplotype analyses, data were phased using shapeit2 (Delaneau and Marchini 2014; Delaneau et al. 2013). The R package rehh was used to calculate haplotype statistics within and between groups of interest. An increase in haplotype homozygosity is expected in regions where selection has occurred (Gautier and Vitalis 2012). The integrated haplotype homozygosity score (iHS) was calculated within resident, migratory, *H. p. purpureus* and *H. p. californicus* groups separately. iHS is used to detect positive selection by identifying areas with increased haplotype homozygosity. Cross‐population extended haplotype homozygosity (xpEHH) was calculated between resident and migratory groups and between the two subspecies. xpEHH identifies regions where haplotype homozygosity is significantly increased in one of the groups. iHS and xpEHH and the corresponding −log_10_(*p*) values were visualised in Microsoft Excel. The *p*‐values were corrected using the Benjamini‐Hochberg method with a 1% false discovery rate (Benjamini and Hochberg 1995). Genes in or adjacent to regions with peaks in haplotype statistics were identified using the annotated reference genome.

## 3 Results

### 3.1 DNA Sequencing and Filtering

The fastqc results did not suggest any significant errors during sequencing. After processing the raw sequence reads and filtering the data, 76,154 SNPs and 43 individuals remained for population analyses.

### 3.2 Population Analyses

*F*_ST_ values ranged from 0.0068 to 0.4522 and were highest between the two subspecies groups. After Benjamini‐Hochberg corrections, 14 of the 28 comparisons were significant. All but three significant comparisons were between the two subspecies (Table 2). The remaining significant values were between SCA and WA, CBC and NS and CBC and LAB.

**TABLE 2:** Pairwise *F*_ST_ comparisons using 76,154 SNPs for the eight populations.

	SCA	OR	WA	VI	CBC	NS	LAB	NL
SCA		0.0785	**0.0321**	0.0750	**0.0107**	**0.0464**	0.0714	**0.0357**
OR	0.0550		0.0571	0.0929	**0.0071**	**0.0393**	0.0679	**0.0429**
WA	**0.0347**	0.0232		0.0536	**0.0036**	**0.0179**	**0.0500**	**0.0250**
VI	0.0510	0.0290	0.0119		**0.0143**	0.0642	0.0893	0.0607
CBC	**0.3688**	**0.3829**	**0.3906**	**0.3715**		**0.0286**	**0.0214**	0.1000
NS	**0.4057**	**0.4112**	**0.4208**	0.3932	**0.0094**		0.0821	0.0964
LAB	0.4473	0.4445	**0.4522**	0.4225	**0.0215**	0.0220		0.0857
NL	**0.4166**	**0.4202**	**0.4279**	0.4029	0.0068	0.0100	0.0214	

*Note:* *F*_ST_ values are below the diagonal, and the corresponding *p*‐values are above the diagonal. *F_ST_* values are colored with higher values in red and lower values in green. All bolded values were statistically significant after Benjamini‐Hochberg corrections.

The highest observed heterozygosity was found in VI (0.41) and the lowest in CBC (0.19) (Table 1). The remaining populations had observed heterozygosities between 0.29 and 0.40. Observed heterozygosity and expected heterozygosity did not differ by more than 0.03 in any study population.

**FIGURE 2:**
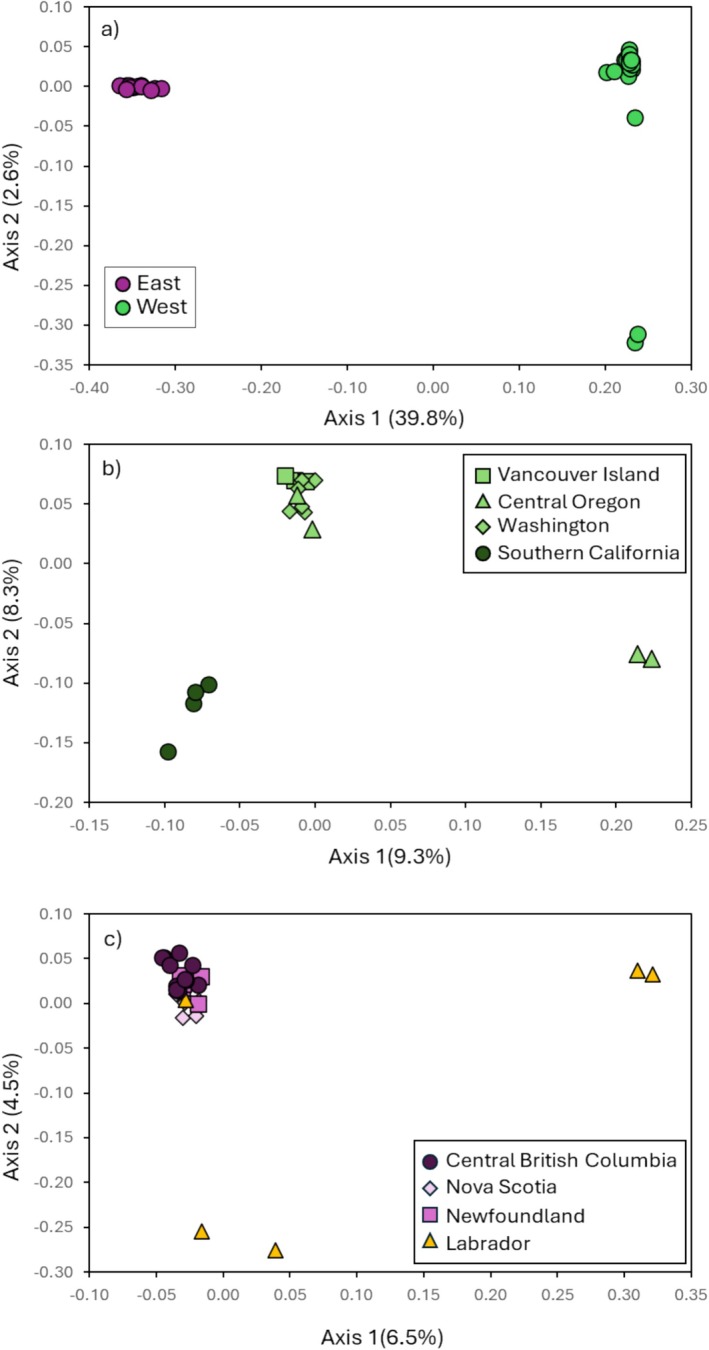
Principal coordinate analysis examining (a) genetic differences between the two subspecies *H. p. purpureus* (*n* = 26; in purple circles) and *H. p. californicus* (*n* = 17; green circles); (b) genetic differences within *H. p. californicus*; individuals from southern California are genetically distinct from all other *H. p. californicus* populations; and (c) genetic differences within *H. p. purpureus* which forms a single panmictic genetic clusters. All three plots were analyzed with 76,154 SNPs.

The PCoA using all individuals showed the strongest clustering along the first axis (39.8%), grouping individuals together according to subspecies (Figure 2a). The second axis explained 2.6% of the variance and separated several *H. p. californicus* individuals from Central Oregon. The PCoA with just the *H. p. californicus* group showed three clusters along the first PC (9.3%) SCA, two Central Oregon birds and the remaining sampling sites. Further separation was found along the second PC (8.3%) (Figure 2b) with SCA and two individuals from OR separating from the remainder of the individuals (VI, WA, OR). Within the *H. p. purpureus* group, the PCoA showed weak clustering along the first axis (6.5%). CBC, NL and NS populations were clustered together with some mixing of individuals and the LAB population although some LAB individuals were quite different (Figure 2c). The second axis (4.5%) separated two LAB individuals.

**FIGURE 3:**
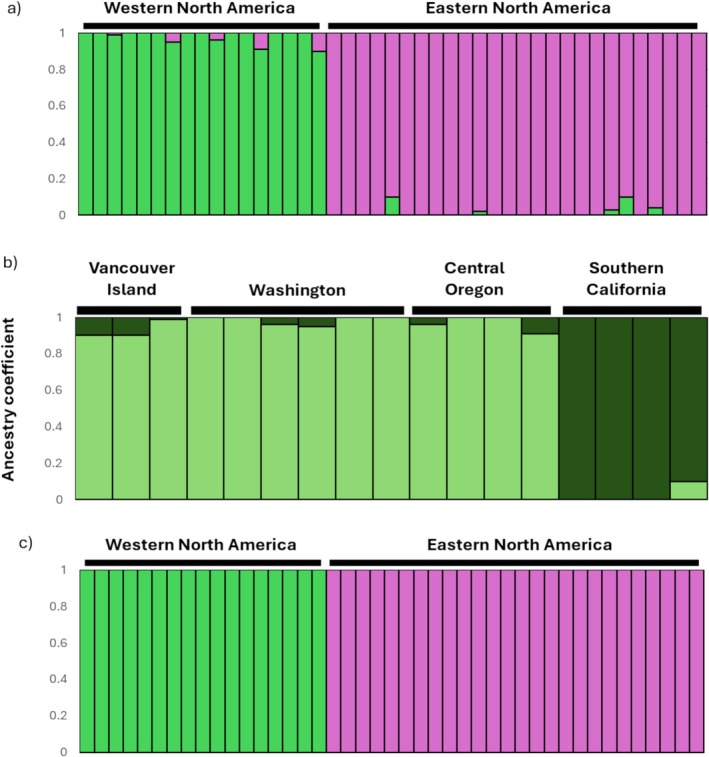
Clustering analysis examining (a) genetic differences between the two subspecies *H. p. purpureus* (*n* = 26; in purple) and *H. p. californicus* (*n* = 17; in green) as evaluated with LEA. *K* = 2 was the best supported model for this analysis and recognised the two subspecies as distinct genetic clusters. (b) genetic differences within the *H. p. californicus* (*n* = 17) subspecies as evaluated with LEA. *K* = 2 was the best supported model for this analysis, and individuals from southern California formed a distinct genetic cluster from all other individuals from west coast populations of North America. (c) Individual ancestry coefficients for the two subspecies *H. p. purpureus* (*n* = 26; in purple) and *H. p. californicus* (*n* = 17; in green) as evaluated with DAPC. *K* = 2 was the best supported model for this analysis and recognised the two subspecies as distinct genetic clusters.

LEA suggested that the best value of *K* was two. The ancestry matrix showed individuals split into their corresponding subspecies (Figure 3a). Higher values of *K* did not show consistent clustering of individuals when using all individuals. Within the *H. p. californicus* subspecies at *K* = 2, the SCA group clustered separately from the other three populations (Figure 3b). The ancestry matrices using the *H. p. purpureus* subspecies did not show any clear clustering at any of the *K* values.

DAPC detected genetic between the two subspecies and indicated that *K* = 2 (Figure 3c) was the strongest supported group (BIC = 395) matching the results of LEA. When we analyzed genetic structure within both subspecies, the software indicated that *K* = 1 was the best supported model of genetic clustering within both *H. p. californicus* and *H. p. purpureus*.

### 3.3 Genome Scans

**FIGURE 4:**
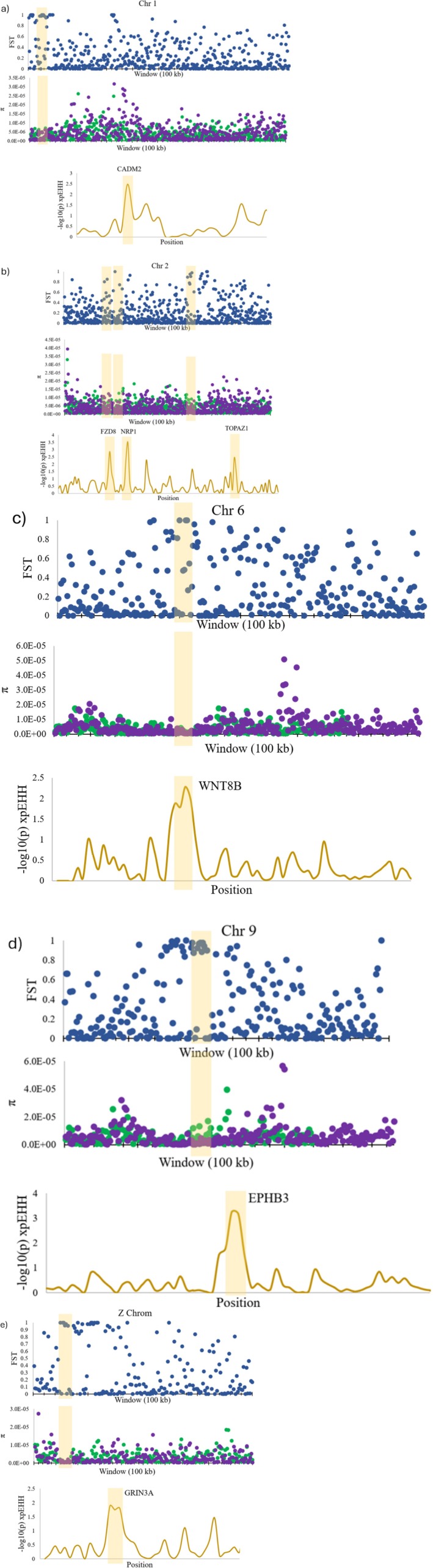
Genome scans comparing subspecies including *F*_ST_ *F*_ST_, π and −log_10_(*p*) xpEHH. The π plots show both subspecies; *H. p. purpureus* (purple) and *H. p. californicus* (green). Highlighted regions contain genes of interest located in regions with the highest *F*_ST_ values, lowered π values in at least one population and significant *p*‐values after corrections from xpEHH. (a) A 55,000 Mb region of chromosome 1 including the region associated with the CADM2 gene. (b) A 70,000 Mb region of chromosome 2 including the regions associated with the FZD8, NRP1 and TOPAZ1 genes. (c) A 30,000 Mb region of chromosome 6 including the region associated with the WNT8B gene. (d) A 25,000 Mb region of chromosome 9 including the region associated with the EPHB3 gene. (e) A 20,000 Mb region of the Z chromosome including the area containing the GRIN3A gene.

The *F*_ST_ genome scan comparing the two subspecies resulted in a mean weighted *F*_ST_ value of 0.3796 across the genome. The chromosomes that had windows with *F*_ST_ values in the top 99.9th percentile in conjunction with lowered/elevated π values were chromosomes 1, 1A, 2, 4, 6, 8, 9, 15 and the Z chromosome (Figure 4). The haplotype comparison using xpEHH showed distinct peaks above a −log_10_(*p*) of 2.5 and after corrections it was confirmed that −log_10_(*p*) values > 2.0 were considered significant. This value was therefore used as the threshold for identifying outlier xpEHH regions on each chromosome. Over 30 genes within or next to these regions were identified (Table 3). Genes of interest included genes related to brain and behaviour such as FZD8, NRP1, WNT8B and GRIN3A (Boyd et al. 2015; Garda et al. 2002; Horton et al. 2020; Schubert et al. 2000; National Center for Biotechnology Information (NCBI) 2022). Other genes were associated with sexual differentiation (TOPAZ1), and feather development (EPHB3) (Backström et al. 2013; Baillet et al. 2011; Matsunaga and Okanoya 2009; Olson et al. 2015).

**TABLE 3:** Genes of interest identified as putatively under selection.

Gene	Chromosome	Function	Comparison identified in
CADM2	1	Associated with environmental adaptation; in chickens they are found to play a role in energy metabolism and whole body energy homeostasis (Xie et al. 2026)	Subspecies and Migratory vs. Resident
TOPAZ1	2	Sexual differentiation (Baillet et al. 2011)	Subspecies and Migratory vs. Resident
GRIN3A	Z	Glutamate receptor gene associated with neuronal development and potentially involved in cognitive phenotypes of vertebrates (O'Rourke and Boeckx 2018; Horton et al. 2020)	Subspecies and Migratory vs. Resident
FZD8	2	Associated with embryogenesis and neural development (Boyd et al. 2015)	Subspecies
NRP1	2	Associated with embryonic vascular development and nervous system patterning Matsunaga and Okanoya (2009); Olson et al. (2015)	Subspecies
WNT8B	6	Associated with neural development and forebrain patterning (Garda et al. 2002)	Subspecies
EPHB3	9	Associated with feather morphogenesis, and neural development (Suksaweang et al. 2012)	Subspecies
MPZL1	1	Song development during juvenile song learning phase (Olson et al. 2015)	Migratory vs. Resident
GRIK1	1	Song development during vocal learning phase (Wang et al. 2019)	Migratory vs. Resident
RCSD1	1	Flanking region associated with ghrelin regulation and production (Tine et al. 2016)	Migratory vs. Resident
LYPLAL1	3	Associated with lipid accumulation and transport (Cohen et al. 2011)	Migratory vs. Resident
PKN2	8	Expressed in hindbrain (Garcia‐Concejo and Larhammar 2021)	Migratory vs. Resident

*Note:* Gene name, chromosome location, gene function and the level of comparison (at the subspecies level or migratory behaviour level) that identified the gene as putatively under selection.

**FIGURE 5:**
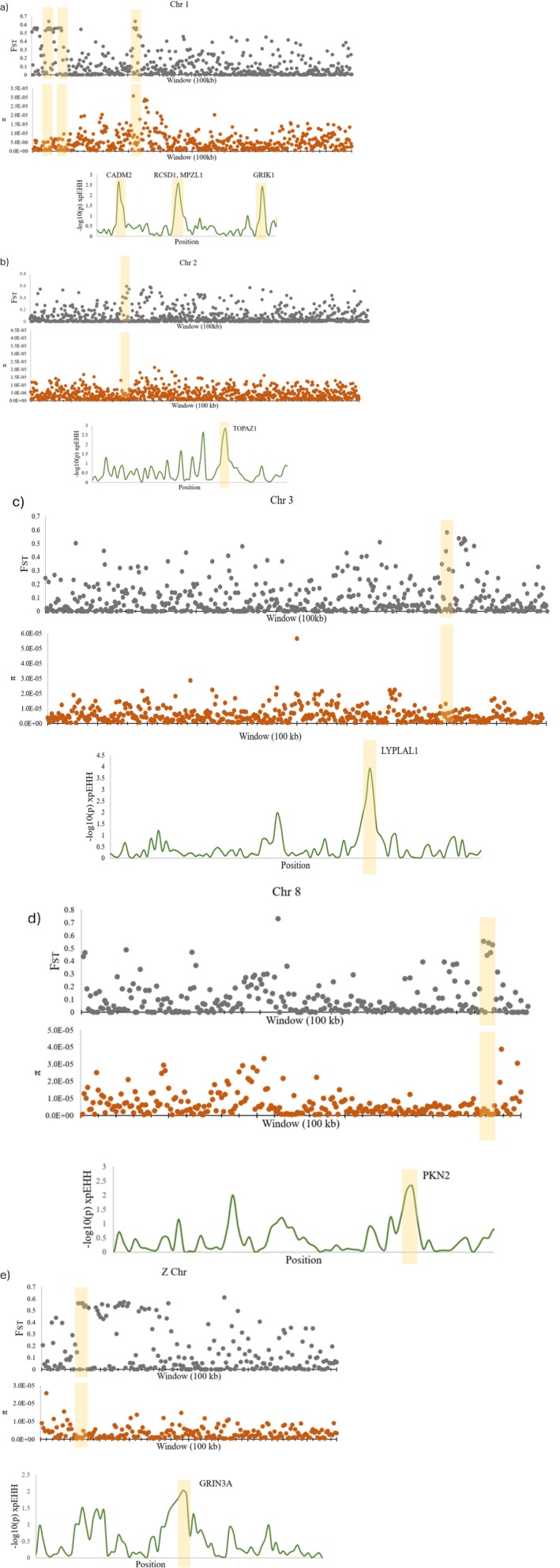
Genome scans of migratory and resident comparisons showing *F*_ST_, π and −log_10_(*p*) xpEHH. The π plots depict the nucleotide diversity in the migratory populations. Yellow highlighted regions contain genes of interest located in regions with the highest *F*_ST_ values, lowered π values and significant *p*‐values from xpEHH. (a) A 50,000 Mb region of chromosome 1 including the regions associated with the CADM2, RCSD1, MPZL1 and GRIK1 genes. (b) An 80,000 Mb region of chromosome 2 including the region associated with the TOPAZ1 gene. (c) A 50,000 Mb region of chromosome 3 including the region associated with the LYPLAL1 gene. (d) A 25,000 Mb region of chromosome 8 including the region associated with the PKN2 gene. (e) A 20,000 Mb region of the Z chromosome including the area containing the GRIN3A gene.

Between resident and migratory populations, the mean weighted *F*_ST_ value from the genome scan was 0.1676. Chromosomes 1–3, 8 and the Z chromosome were used to calculate haplotype statistics (Figure 5). After corrections it was determined that a −log_10_(*p*) xpEHH of > 2.1 was considered significant and therefore used to identify differentiated regions between the migratory phenotypes. Three of the genes found in significantly differentiated regions were also identified in the subspecies comparison (CADM2, TOPAZ1, GRIN3A) (Table 3). In addition, five other genes were identified related to: song (MPZL1 and GRIK1), hunger (ghrelin hormone RCSD1), fat development (LYPLAL1) and brain function (PKN2) (Olson et al. 2015; Wang et al. 2019; Tine et al. 2016; Cohen et al. 2011; Garcia‐Concejo and Larhammar 2021).

## 4 Discussion

### 4.1 Population Structure

Our genetic analyses with ddRADseq data revealed that the largest genetic differences are between the two subspecies of purple finch. Genetic differences between subspecies likely reflect that the two subspecies likely resided in separate glacial refugia during the LGM and genetic differences likely arose due extended periods of isolation. Eastern and western genetic splits are widely seen across diverse taxa and reflect the lasting effects that Pleistocene glaciations had on genetic patterns (Burg et al. 2014; Knowles 2001; Macfarlane et al. 2016; Weir and Schluter 2004). Our analyses of migratory and resident populations found low genetic differentiation within the *H. p. purpureus* subspecies and the presence of genetic panmixia reported in this study suggests that gene flow between resident and migratory populations is higher than we predicted. Despite genetic panmixia in the *H. p. purpureus* subspecies, we did identify genes under selection associated with pre‐migratory and migratory behaviour and physiology; thereby indicating that genetic differences exist between migratory and resident populations.

Macfarlane et al. (2016) suggested *H. p. californicus* resided in a refugium in the Pacific Northwest, citing lowered genetic diversity in SCA in comparison to more northerly populations. In our study, VI had the highest observed heterozygosity in the *H. p. californicus* group, supporting that the refugium was closer to the northern extent of their range and populations expanded southward as the ice melted (Table 1). It has been suggested that a glacial refugium existed that connected Vancouver Island and coastal British Columbia due to the lowered sea levels during the last glacial period (Byun et al. 1997; Pruett et al. 2013). Both the previous study and the current study support the presence of two glacial refugia for the purple finch, with at least one likely being in the Pacific Northwest (Macfarlane et al. 2016).

When examining fine‐scale population structure within the *H. p. californicus* subspecies, we consistently found that the SCA individuals separated from other populations (Figures 2 and 3), suggesting that gene flow between SCA and other west coast populations is reduced. The previous study with microsatellites found OR and SCA grouped together, however, large SNP datasets such as the one used in this study may uncover different fine‐scale population structure (Hodel et al. 2017; Jeffries et al. 2016; Lemopoulos et al. 2019; Sunde et al. 2020; Zimmerman et al. 2020). The SCA samples in this study are specifically from the San Bernardino National Forest region. Other forest bird species such as the mountain chickadee ( *Poecile gambeli* ) also show genetic differentiation in this southern California area (Hindley et al. 2018; Manthey et al. 2012; Spellman et al. 2007). Habitat fragmentation in this area may be driving genetic differentiation in both avian and non‐avian taxa as tree‐free basins separate the north from the south (Hindley et al. 2018; Tan and Wake 1995; Wake 1997). Additionally, it is a well‐documented genetic pattern that populations on the peripheries of a species' range are more likely to be fragmented and more sparsely populated, leading to genetic isolation (Botero‐Delgadillo et al. 2017; Mendelsohn et al. 2020; Rossum et al. 2003).

Within the *H. p. purpureus* subspecies, little population structure was observed in the PCoA (Figure 2c). There are likely significant levels of gene flow between populations as other analyses did not suggest distinct separation within this subspecies. The previous study also suggested high levels of mixing within the *H. p. purpureus* subspecies, particularly in CBC (Macfarlane et al. 2016). While geographic distance is likely contributing to the population structure, it is important to note that CBC and the closest population sampled to the east (NS) are approximately 4000 km apart which may exaggerate structure shown in the PCoA and why several LAB individuals separate from the main clusters. Obtaining samples from sites across the rest of its range (in Alberta, Saskatchewan, Manitoba, Ontario and Quebec) would be beneficial to fully understand the genetic patterns within the *H. p. purpureus* subspecies.

### 4.2 Genome Scans Between Subspecies Groups

The genome scans between the two subspecies showed numerous areas of differentiation and potential divergent selection. A large number of differentiated regions may be expected considering their prolonged isolation during the Pleistocene. One of these differentiated regions was located on chromosome 2 and included the NRP1 gene. NRP1 has been implicated in the evolution and development of bird song (Matsunaga and Okanoya 2009; Olson et al. 2015). This may be of particular importance as song is critical in sexual selection and may act as a reproductive barrier between the subspecies. The two subspecies are noted to have differing songs, with the *H. p. purpureus* song being more varied (Wootton 2020). TOPAZ1 is another gene that may influence sexual selection or sexual dimorphism, as it is differentially expressed between sexes (Baillet et al. 2011). Sexual differentiation and dimorphism contribute to reproductive barriers, causing groups to diverge from one another over time due to sexual selection both within and between the sexes.

In addition to differences in song, it has been suggested that other behavioural differences may exist between the subspecies (Wootton 2020; Sibley 2014). Although not well studied, it is suggested that individuals in the West have different nest location preferences than those in the East (Audubon 2022). We found five genes that may have undergone a selective sweep. These genes are related to brain development, behaviour, or memory (FZD8, WNT8B, CADM2, GRIN3A, EPHB3). These genes may play a role in observed behavioural differences between the subspecies. Further, the song differences between the subspecies could be influenced by some of these cognitive factors which would help further maintain genetic differences. In other bird species, it has been found that differences in behaviour can be explained by genetics, even when populations are readily interbreeding (Pravosudov et al. 2012). Different brain morphology and behaviour are often associated with specific regions of the genome and can be influenced by selective pressures, indicating that behavioural differences can reflect genetic divergence (Branch et al. 2022; Pravosudov et al. 2012).

### 4.3 Genome Scans Between Migratory Phenotypes

Between migratory and resident populations, three genes were identified that have a role in memory, behaviour, or plasticity. These genes included GRIN3A, CADM2 and PKN1 (Garcia‐Concejo and Larhammar 2021; Horton et al. 2020; National Center for Biotechnology Information (NCBI) 2022). It is not surprising that genes related to behaviour and memory are potentially associated with migratory behaviour, as migration has a known genetic component that can vary from species to species (Delmore et al. 2020, 2016; Lundberg et al. 2017; Parody‐Merino et al. 2019; Peterson et al. 2013). We did not find any overlap between the genes found in this study and genes found in other studies of migration.

LYPLAL1 was another gene that was associated with increased differentiation and extended haplotype homozygosity, suggesting it may have been under differing selection between the two migratory phenotypes (Figure 5c). This gene has been associated with the accumulation of lipids in the liver (Cohen et al. 2011). Migratory birds experience an increase in food consumption and fat stores prior to migration, and it is not surprising that a gene such as LYPLAL1 may show differential selection between migratory and resident individuals (McCabe and Guglielmo 2019). Related to an increase in fat stores, the RCSD1 gene (Figure 5a) is thought to flank a region related to the production of ghrelin, a hormone involved in food intake and stimulating appetite (Tine et al. 2016). This hormone has been associated with differences in migratory behaviour in many other bird species such as garden warblers ( *Sylvia borin* ), yellow‐rumped warblers ( *Setophaga coronata* ) and common blackbirds ( *Turdus merula* ) (Eikenaar et al. 2018; Lupi et al. 2022; Goymann et al. 2017).

Three other genes (TOPAZ1, MPZL1 and GRIK1) were related to sexual differentiation or sexual selection, particularly in influencing song development (Baillet et al. 2011; Liu et al. 2013; Olson et al. 2015). Migratory behaviour often differs between sexes, with males migrating earlier than females to arrive at the breeding grounds sooner (Maggini and Bairlein 2012; Kissner et al. 2003). These genes may be of interest in the migratory phenotypes due to differences in migration commonly observed between sexes.

### 4.4 Shared Genes of Interest

The *F*_ST_ values from the subspecies genome scans were overall higher than the values comparing resident and migratory populations, indicating that there is stronger differentiation between subspecies than between migratory phenotypes. Interestingly, three genes were identified as being putatively under selection in both the subspecies and migratory/resident comparisons. CADM2 and GRIN3A are both associated with learning, memory and behavioural plasticity (Figures 4a,e and 5a,e) (Horton et al. 2020; National Center for Biotechnology Information (NCBI) 2022). The *H. p. californicus* subspecies is primarily resident while *H. p. purpureus* contains many migratory individuals, suggesting that some of the genes identified in the subspecies analyses may also influence migratory behaviour (Wootton 2020). While many of these genes may not be fully understood in birds, it is possible that these genes are more variable, allowing selection to act upon these variants more often. The third gene found in both comparisons is the TOPAZ1 gene related to sexual differentiation (Figures 4b and 5b) (Baillet et al. 2011). There are likely sex differences in the purple finch's migratory behaviour, a common phenomenon observed in birds. Additionally, purple finch are sexually dimorphic species with additional differences between sexes between the subspecies (Wootton 2020). The aforementioned genes may be of particular interest in the purple finch due to their apparent association with multiple processes.

## 5 Conclusions

This study has provided additional support for the current genetic groupings of purple finch, as our findings depict similar patterns as previous studies using different molecular markers. The distinct subspecies split is representative of a long period of isolation in separate glacial refugia during the last glacial maximum. Within the *H. p. californicus* subspecies we have identified SCA population as being genetically distinct due to fragmented habitat may be of special conservation concern. It will be important to monitor this population in the future. To fully understand the fine‐scale population structure within the *H. p. purpureus* subspecies, samples from between CBC and NS will need to be analyzed. Utilising thousands of SNPs has allowed for more in depth comparisons of both the subspecies and migratory/resident populations. We have identified numerous genes that may be responsible for some of the differentiation observed between the *H. p. californicus* and *H. p. purpureus* subspecies.

## Author Contributions

**Danika Schramm:** formal analysis (lead), writing – original draft (lead). **Theresa M. Burg:** conceptualization (lead), funding acquisition (lead), resources (lead), supervision (lead), writing – review and editing (lead).

## Funding

This work was by the Natural Sciences and Engineering Research Council of Canada Discovery Grant (RGPIN 2019 05068).

## Conflicts of Interest

The authors declare no conflicts of interest.

## Data Availability

The data analyzed for the manuscript are openly available in The Federated Research Data Repository at https://doi.org/10.20383/103.01019.
